# A Delphi study to construct a CanMEDS competence based inventory applicable for workplace assessment

**DOI:** 10.1186/1472-6920-12-86

**Published:** 2012-09-14

**Authors:** Nele RM Michels, Joke Denekens, Erik W Driessen, Luc F Van Gaal, Leo L Bossaert, Benedicte Y De Winter

**Affiliations:** 1 Skills Lab, Faculty of Medicine and Health Sciences, University of Antwerp, Campus Drie Eiken – D.R.314 – Universiteitsplein 1, Antwerp 2610, Belgium; 2 Educational Department and Faculty of Medicine and Health Sciences, University of Antwerp, Middelheimcampus – M.A.211 – Middelheimlaan 1, Antwerp 2020, Belgium; 3 Department of Educational Development and Research, Faculty of Health, Medicine and Life Sciences, Maastricht University, PO Box 616, Maastricht 6200MD, The Netherlands; 4 Department of Diabetology, Metabolism and Clinical Nutrition, Antwerp University Hospital, Wilrijkstraat 10, Antwerp 2650, Belgium; 5 Faculty of Medicine and Health Sciences, University of Antwerp, Campus Drie Eiken, D.S.034 – Universiteitsplein 1, Antwerp 2610, Belgium; 6 Skills Lab, Faculty of Medicine and Health Sciences, University of Antwerp, Campus Drie Eiken, D.T.226 – Universiteitsplein 1, Antwerp 2610, Belgium

**Keywords:** Portfolio, Medical education, Content validity, CanMEDS roles, CanMEDS competences, Workplace assessment, Delphi study, Competence inventory

## Abstract

**Background:**

During workplace based learning students develop professional competences and an appropriate performance. To gain insight in the learning process and to evaluate competences and performance, assessment tools are essential and need to be of good quality. We aimed to construct a competence inventory applicable as an instrument to measure the content validity of workplace based assessment tools, such as portfolio.

**Methods:**

A Delphi study was carried out based on the CanMEDS Roles Framework. In three rounds, experts (N = 25–30) were invited to score the key competences per CanMEDS role on relevance (6-point Likert-scale), and to comment on the content and formulation bearing in mind its use in workplace based assessment. A descriptive analysis of relevances and comments was performed.

**Results:**

Although all competences were scored as relevant, many comments pointed at a lack of concrete, transparent and applicable descriptions of the key competences for the purpose of assessment. Therefore, the CanMEDS roles were reformulated in this Delphi procedure as concrete learning outcomes, observable and suitable for workplace based assessment.

**Conclusions:**

A competence based inventory, ready for validating workplace based assessment tools, was constructed using a Delphi procedure and based on a clarification and concretisation of the CanMEDS roles.

## Background

For medical doctors, specific roles and competences have been defined in both undergraduate and postgraduate training, as well as in continuing medical education. These roles and competences are classified in frameworks such as the CanMEDS (Canadian Medical Education Directives for Specialists) Roles Framework [1], the six core competences identified and described by the Accreditation Council for Graduate Medical Education (ACGME competencies) [2] and Tomorrow Doctor’s at the UK [3].

During workplace based learning, students develop professional competences and an appropriate performance [4,5]. Insight into students’ performance has to be obtained by assessing their learning processes and their competences at the authentic workplace, using assessment tools of good quality [6]. It is generally accepted that assessing workplace learning is difficult by the use of a single traditional assessment method [7]. Therefore, faculties most often combine different assessment tools such as mini-CEX (mini Clinical Evaluation Exercise), case reports on patient encounters, Case based Discussions (CbD), or multisource feedback, all developed to suit the workplace based context. As the whole spectrum of behaviours and attitudes should be taken into account in, both controlled observed situations and daily practice, the combination of formative and summative assessment is suggested [8-10]. In this view, the use of a portfolio, conveying evidence of learning gathered by students from various sources in various contexts, opens perspectives [11-13].

In this study, we wanted to construct a validated inventory to investigate whether clinical competences could really be assessed by a workplace based assessment tool like portfolio. As such we will be able to measure the content validity of portfolios. Content validity is defined as the degree to which the content of the assessment instrument covers the intended learning objectives. Studies addressing the content validity using validating inventories are scarce in the field of portfolio assessment [14-16] [Michels NRM, Remmen R, Denekens J, van Rossum H, De Winter BY. A systematic review of validity facets in portfolio research: hit the target and don’t miss the point. Submitted], but nevertheless necessary to explore the quality of this tool.

To construct a competence inventory, we used a Delphi procedure to obtain expert opinion on the competences medical students can and should gain during workplace learning. In the Delphi procedure the CanMEDS Roles Framework, already introduced in our medical school and validated in an international, European context [17], was used as basis for the inventory.

## Methods

### Delphi procedure background

We used a conventional Delphi survey in a paper-and-pencil form [18,19]. A starting document was designed and sent to a group of respondents. After the document was returned, the data were anonymously analysed and the document was revised. The revised version was then resent to the respondents, including the opinions and remarks of colleague-respondents. Classically, several rounds are organised in a Delphi procedure, ultimately leading to a consensus document. To obtain a scientifically sound Delphi round and to anticipate on unpredictable distractions, some control systems were built in. On behalf of the experts, strict guidelines and clear information regarding the Delphi process and the main research question as well as regarding their particular assignment were written and distributed before each Delphi round. In addition, the principal researcher analysing the various data rigorously guarded the proceeding of the Delphi process towards achieving a consensus. Additional interviews with a small number of the experts were performed to properly guide the process and to discuss whether the original design was maintained. A large group of experts with several backgrounds and, clearly, different rankings and positions participated, offering the ideal audience for a Delphi method. They are described further on in detail. Anonymity was guaranteed.

Since 2002, the medical school at the University of Antwerp uses a portfolio to mentor and assess students during their fulltime internship, organised in year 6 [20,21]. Accordingly, the setting of this study was decided to be the internships during undergraduate medical training, specifically workplace based learning and assessment.

### Delphi expert panel

A group of 30 experts was invited to participate. The experts were selected based on two main criteria: either from the educational staff provided they possessed medical experience, or from the medical staff provided they were familiar with the portfolio as a workplace based assessment tool. In more detail, the expert team consisted of 7 internal staff members (all from the skills lab team), 20 external staff members (being general practitioners (N = 6) or clinicians from 14 different clinical disciplines (N = 14)), and 3 members of the educational staff with medical experience. As we wanted to minimise the bias caused by the fact that portfolios in different settings could have different meanings and could include different contents, we selected experts mostly linked to the University of Antwerp and/or the Antwerp University Hospital. To compensate this locality, one of the inclusion criteria was having international expertise. We discussed the aim and the procedure of the study with the experts and handed over sufficient information. They all signed informed consent. Ethical approval was given by the ethics committee of the Antwerp University Hospital.

### Delphi starting document

A subgroup of the experts designed the starting document. A Flemish translation of the CanMEDS roles [1] was previously agreed at a committee meeting of the 5 Flemish universities [22]. Our group decided to use this as starting point. As such, all the 7 CanMEDS roles, namely Medical Expert, Communicator, Collaborator, Manager, Health advocate, Scholar, and Professional, were listed and structured with each of them having 2 to 7 corresponding key competences (Additional file 1: Table S1 Starting document).

### Delphi protocol – first round

Table 1 illustrates the protocol of our Delphi study. In the first Delphi round, the experts were asked to consider two main issues. Firstly, they were asked to scale the relevance of the key competences bearing in mind assessment of students during their internships. Specifically, they were asked whether the formulation of the key competences was appropriate for assessing observable behaviour at the workplace. The experts scored on a 6-point Likert scale (1 = not relevant; 6 = very relevant), thereby acknowledging that all scores >3 were considered as relevant. Secondly, there was free space for suggestions or remarks per key competence and per CanMEDS role as a whole, both on formulation and on content.

**Table 1:** Protocol description of the Delphi procedure

**Delphi round**	**N experts**	**Response rate**	**Document**	**Task / Question**	**Analysis**
n° 1	30	83% (25/30)	document 1: Flemish translation of CanMEDS (cfr. Additional file 1)	- relevance? (6 point Likert scale)	- frequency
				- suggestions?	- listing suggestions
n° 2	25	88% (22/25)	document 1 + round 1 comments	- relevance? (6 point Likert scale)	- frequency
				- (non)-agreement on listed suggestions?	- listing (non-) agreements
n° 3	25	96% (24/25)	document 2: revision of document 1 using round 1 & 2 comments	1) Are the competences formulated sufficiently concrete and assessable?	- last revision
				2) Is there overlap between certain competences and/or roles?	
				3) Are certain aspects of competences or roles missing?	

Description of the three Delphi rounds concerning the number of invited experts, the response rate, the document in that particular round, the requested task of the experts and the analysis performed.

### Delphi data analyses

Descriptive statistics (medians, the 25 and 75^th^ percentiles and the percentage of non-relevant scores) were calculated after every round. A Mann–Whitney *U* test was performed to investigate potential different scoring behaviours between experts of the internal staff and external experts.

All the suggestions and remarks were anonymously and literally registered. For the purpose of a final analysis, at the end of the Delphi study, they were structured and categorised based on the principles of thematic analysis (by NM) [23].

### Delphi protocol – second round

In the second Delphi round the previous analyses on the frequencies and remarks, were forwarded anonymously to all the respondents of the first round (N = 25). Equipped with the feedback, the experts had to score once again all the key competences on their relevance taking into account the medians and the remarks of all the experts. Besides, they were asked to formulate again suggestions and remarks.

### Delphi protocol – third round

After the first two Delphi rounds, a thorough revision of the competence inventory was carried out in line with the remarks and suggestions of the experts, the analyses of the previous rounds, literature data, and discussions with some of the experts. This revised version of the inventory was sent in the third Delphi round to the 25 experts of the second round. The experts were required to give remarks and/or suggestions keeping in mind the following 3 questions: 1) Are the key competences formulated sufficiently concrete and assessable as regards the workplace? 2) Is there overlap between different key competences within or over specific CanMEDS roles? and 3) Are certain aspects of the key competences or CanMEDS roles still missing?

A fourth and last Delphi round was held to assure all the experts agreed with the consensus reached at the end of the third Delphi round.

## Results

Twenty-five of the 30 experts responded in the first Delphi round (response rate of 83%) (Table 1). Three non-responders withdraw their participation due to a lack of time, one non-responder estimated himself not sufficiently competent in this field and one expert did not respond at all. The second Delphi round had a response rate of 88%: from the 25 responders of Delphi round 1, 22 responded in the second round. At this time point, the non-responders were unable to take part due to medical reasons or a lack of time in the proposed time period. Nevertheless, they all agreed to participate in subsequent rounds. Therefore, the 25 responders of round 1 were mailed for cooperation in the third Delphi round. Only 1 expert could not participate in the third round because of medical reasons, resulting in a response rate of 96%.

### Delphi round 1

**Figure 1:**
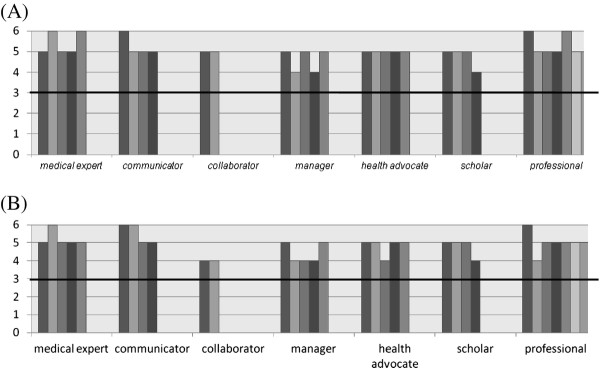
**The relevances of the key competences per CanMEDS role during A) Delphi round n°1 and B) Delphi round n°2, represented as the median score.** Scores above 3 (on a Likert scale of 6) indicate that the experts score this key competence as relevant.

The median scores on relevance of the key competences demonstrated that all the key competences of all the different roles were rated as relevant (Figure 1A and Additional file 1: Table S1). Five key competences reached 6/6 as median score, while the lowest median score was still 4/6 for 3 key competences (Additional file 1: Table S1). Although these median scores underline the relevance of all key competences globally, some experts scored some key competences as non-relevant. Key competence n° 12, 13, 15, and 25 were scored non-relevant by respectively 25.1, 32, 28, and 36.3% of the experts (Additional file 1: Table S1). The Mann–Whitney *U* test found no statistical significant (p = 0.581) differences between the internal and the external expert group.

Interestingly, the first Delphi round delivered many remarks and suggestions (N = 389). The number of remarks per key competence, per CanMEDS role in general, and on the list as a whole are presented in Additional file 1: Table S1.

Categorisation in themes showed that most of the comments regarded the applicability *for assessment* of the key competence (33%), and the lack of concreteness of the competence descriptions (32%). Furthermore, the experts suggested additional terms or concepts concerning both the description and the content of the key competences (11%), and mentioned overlaps between key competences or CanMEDS roles (5%). Table 2 depicts some representative quotes. The importance of key competences or CanMEDS roles was additionally confirmed by the remarks (8%), and the connection between some key competences and the (undergraduate) educational level of the students was questioned (8%). Three percent of the remarks dealt with comments on the general education in medical school and on the internships itself or their organisation.

**Table 2:** Examples of quotes given by the experts in the first Delphi round

**comments**	**example of quotes**	**role & n° of key competence**
**about applicability for assessment**	*“What exactly do you want to assess?”*	Medical Expert – n°5
	*“Isn’t it better to evaluate whether students ask for supervision in time or not?”*	Professional – n° 30
**on the lack of concrete competences**	*“What do you mean by ‘additional information’?”*	Collaborator – n°11
	*“too vague and formulated too difficult”*	Health advocate – n° 18
**on formulation or content**	*“communication ****with ****the patient is missing (a bit)”*	Communicator – general remark on the role
	*“+ responsibility (daring to give and to take)”*	Collaborator – general remark on the role
**on overlap**	*“Isn’t this rather a key competence of Communicator?”*	Scholar – n° 25

### Delphi round 2

As seen in Figure 1B, 8 key competences scored a median of 4 on the Likert scale compared to 3 key competences in round 1. The 3 key competences, mentioned in round 1, remained at a score 4 in the second round. Additionally, 2 key competences of the Collaborator role (n°10 and n°11), 1 extra key competence of the Manager role (n°14), 1 of the Health advocate role (n°19), and 1 of the Professional role (n°27) scored lower on relevances than in round 1. For the highest scores, 3 key competences scoring a median of 6 were identical with the first round. There was 1 additional score of 6 in the Communicator role (n°7). Nevertheless, both in the Medical Expert role and the Professional role 1 competence scored 5 instead of 6 (n°5 and n° 30).

The Mann–Whitney *U* test found no statistical significant (p = 0.428) differences between the internal and the external expert group.

In both rounds, the highest scores on relevance of key competences are found in the Medical Expert role, the Communicator role, and the Professional role, while the lowest (still relevant) scores are seen in the Collaborator, the Manager, the Health Advocate and the Scholar role.

In the second Delphi round experts were asked to react on their colleagues’ remarks during the first Delphi round. The huge amount of remarks made it impossible to take decisions on including or excluding remarks on the base of percentages. Sometimes opinions were different and contradictory, sometimes not. Besides, a lot of remarks were too fundamental to reject, although some made by a minority of experts. Based on the thematic analysis of round 1 and the results of round 2, three main and important issues arose i.e. the need to 1) concretise the competences, hence to formulate them more applicable for assessment purposes, 2) eliminate the existing overlaps between key competences and CanMEDS roles, and 3) add missing aspects.

At this point, a revision of the working inventory of competences became inevitable. CanMEDS roles and key competences were revised in order to take the above mentioned issues on concreteness, overlap, and missing key competences into account. This revision was based on all the data of both Delphi rounds, the original CanMEDS descriptions [24], and literature data dealing with identical investigations on medical competences [17,25,26]. Discussions with some of the experts (BDW and JD) facilitated the process, especially when controversies in the experts’ opinions arose.

### Delphi round 3

In the third Delphi round this adapted inventory was sent to the experts. The purpose was to obtain experts’ remarks and/or suggestions regarding the novel formulation of the competences, regarding existing overlap between certain key competences and/or CanMEDS roles, and regarding missing aspects of competences or roles. Seventy-nine percent of the experts had no major remarks on the renewed inventory (per expert ≤ 11 remarks on the whole list with a median of 7 (3–11 (25–75 percentiles)). All the remarks given by the experts were included in the development of the definitive competence inventory which was confirmed by the fourth and last Delphi round. As presented in Table 3, this competence inventory offers for each CanMEDS role a number of actively formulated competences students have to achieve at the workplace.

**Table 3:** The definitive competence inventory as confirmed by the experts in the last Delphi round

**CanMEDS role**	**Key competence**
	
**Medical Expert**	* has insight in required medical knowledge with regard to a clinical problem, i.e.:
the student	∘ applies the acquired knowledge
	∘ applies medical decision making
	* efficiently applies acquired medical skills with regard to a clinical problem
	* accomplishes a health care plan:
	∘ performs a relevant and adequate intake and anamnesis
	∘ performs an efficient physical or other examination
	∘ generates a differential diagnosis
	∘ efficiently gathers, analysis, and interprets data (from anamnesis, physical examination, and technical investigations)
	∘ generates an accurate diagnosis
	∘ presents efficient treatment plans
	* generates an accurate, multidisciplinary health care plan with specific attention for patient’s self care and follow up care
	* defines symptoms of the most common and critical diseases and recognises alarm symptoms (also for differential diagnosis)
	* integrates the different CanMEDS roles
**Communicator**	* clearly and understandable reports a relevant, complete, systematic and accurate intake and anamnesis
the student	* writes reports concerning patients encounters in the medical record and in referral letters to other health care providers
	* can manage a patient record, and clearly and structurally provides (all) the information to other health care providers.
	* verbally reports on patients encounters to other doctors and health care providers
	* communicates scientific research in a clear, complete and structural way
	* communicates during a patient’s encounter according to the rules of good practice
	* establishes (and maintains) an empathic, trustful and ethical doctor-patient relationship and doctor-family relationship
	* reflects on own communication skills and their progression
**Collaborator**	* knows and involves the profile and competences of other health care providers
	* actively takes part in team work
the student	* effectively contributes to the interdisciplinary teamwork concerning patient care, education and research
	* integrates following aspects with regard to team work:
	∘ taking and giving responsibility
	∘ delegating and organising
	∘ giving and taking suggestions to/of other health care providers
	∘ supporting the “chain-of-care” (increasing effective team work)
	∘ coping with conflicts between professionals
	* reflects on teamwork and on respecting the opinions of other team members
**Manager**	* reflects on self-care and the balance between work and personal development (work/private time management)
the student	* ranks information in order of importance and urgency; responsibly prioritises, and motivates priorities (professional time management)
	* correctly and punctually deals with administrative and organisational tasks
	* registers, classifies, and transfers patient related data in an effective (and trustful) way
	* uses information technology to:
	∘ optimise patient care and practice organisation – (patient related databases)
	∘ stimulate “life long learning” – (medical databases)
	* can work within the health care system and other care systems (welfare, justice) in Belgium
	* has insights in costs of medical care and their implication for society, patients and medical doctors
	* has insights in procedures for solicitations and contractual negotiations
**Health advocate**	* reflects on: psychological, social, economical, biological, ethical, cultural, and religious aspects influencing patients’ health
the student	* attends to the individual patient and the population regarding health-related aspects (primary prevention)
	* deals with prevention and health promotion for the individual patient and the population (secondary prevention)
	* has attention for patient safety
	* efficiently accompanies patients through the health care system and reasons in support of a decision making
	* prioritises the patient’s benefits
	* involves and facilitates the accessibility of health care during daily practice, especially for vulnerable groups of patients
	* reflects on critical incidents in doctor’s practice
**Scholar**	* poses relevant, practical and scientific questions with regard to patient care
the student	* performs searches in medical scientific databases/sources in an efficient, purposeful and rapid way
	* questions the quality of consulted medical scientific databases/sources
	* adequately applies scientific information in decision making in doctor’s practice
	* development and follow up of a personal learning plan
	∘ can critically reflect on daily performance in the doctor’s practice
	∘ describes and analyses own personal learning needs
	∘ applies an adequate learning method
	∘ self evaluates or evaluates with peers his learning results and remediates
	* assists in creating, spreading, and applying new medical knowledge and practice
	* stimulates training of patients, family, students, trainees, other health care workers, population
	* adapts his functioning to societal evolutions in health care
	* is open-minded towards “life long learning”
**Professional**	* utilises the highest quality of care for his patient in an integral, upright and ethical way
the student	* understands the meaning of and applies:
	∘ professional codes
	∘ ethical codes and dilemmas (= uses an ethical frame)
	∘ legal codes
	* reflects on
	∘ own behaviour
	∘ own professional attitude: shows willingness to offer medical care in an optimal, ethical, and patient centred way
	∘ attitude and behaviour of others and evaluates this for himself
	∘ legal implications of patient care (patient rights, professional secrecy or professional confidentiality, DNR-codes, end-of-life coaching)
	∘ professional, ethical and legal codes
	* has an appropriate professional attitude and behaviour, demonstrating
	∘ honesty
	∘ integrity
	∘ engagement
	∘ respect
	∘ understanding, empathy
	∘ altruism
	and remediates (himself) when needed
	* recognises his own limits, weaknesses or lacunas and can cope with these

## Discussion

The goal of this study was to develop an adequate tool to evaluate whether competences can be measured (content validity) by workplace based assessment tools. We used a Delphi procedure to develop a competence inventory based on the CanMEDS roles. Our first aim was to investigate which of the CanMEDS competences could remain or could not remain in this competence evaluation tool. Rather unexpectedly, some fundamental issues arose which will be discussed below.

Firstly, this Delphi study reinforces the CanMEDS Roles Framework based on the high percentages of relevance of the different roles and competences. However, the applicability as an assessment tool for workplace based learning was questioned in this Delphi procedure.

Regarding the relevance of the roles and competences, Ringsted et al. (2006) found comparable mean ratings in a similar study investigating the importance of the aspects of competences described by the CanMEDS roles outside Canada in Denmark: overall mean rating of 4.2/5 versus our 4.8/6 and 4.6/6 in the 1^st^ and 2^nd^ round [17]. In further agreement with our findings, the Communicator role achieved high ratings; whereas the Collaborator and Health advocate role scored rather lower, yet still relevant. In our study, however, the experts perceived the Manager and Scholar role equally relevant as the Collaborator and Health advocate role. Conceivably, this could be explained by the difference in setting: Ringsted et al. surveyed both interns as postgraduate trainees and specialists where the postgraduate trainees and specialists (i.e. more experienced clinicians) perceived the Manager role as more relevant than the group of interns (students).

A second apparent finding was the necessity to reformulate and rearrange the list of the CanMEDS roles and their key competences. The wide and international introduction of the CanMEDS Roles Framework in medical education, shows how valuable they are for outlining the competences students have to achieve to become good doctors. However, the link with assessment is not automatically achieved and was also not intended in the original Framework. In literature, the lack of tools to evaluate students in their attempt to acquire the different CanMEDS roles is acknowledged [27]. Interestingly, the data of our Delphi study strongly emphasised this need. Changes in formulations and structure were however required to obtain a list of key competences formulated in a useful way for assessment at the workplace. In the next paragraph, we will describe the experts’ suggestions to improve the inventory for assessment purposes.

First of all, the experts indicated the need for adequate descriptions and transparent formulations, so that different interpretations of words and expressions could be avoided. Questions such as “to whom?”, “which?”, “what is …?”, “in which way?” need clarifications and vague words like “appropriate” should be specified, for example “in conformity to the proposed rules” [28]. Furthermore, the study confirmed the benefit of the use of active words and phrases, which are more functional for assessment. For example the use of “analyses data” instead of “can handle data”.

Finally, the elimination of overlap was concluded to be necessary. Separating the CanMEDS roles and key competences makes them more usable for assessment offering clear guidelines to students and evaluators [29]. Additionally, quality assurance of medical education programs emphasises a clear link between required outcomes and assessment criteria. This could be controversial, because it creates a possible risk to artificially divide roles and competences, and therefore lead to a fragmentation of performance [30]. In a real and clinical context, overlap does exist and the CanMEDS Roles Framework visually expresses this by overlaying the leaves of the CanMEDS flower. In a recent review Lurie et al. (2009) state that no current measurement tool can assess the ACGME competences (another competence framework) independently of one another [31]. Actually, this points to the well known difficult balance between the necessity for objective assessment of the competences and the existing reality, i.e. how students and doctors perform in the clinical context. In our opinion, both approaches are complementary. We propose our novel, and on research based competence inventory as a tool to assess the different competences by a portfolio in order to prevent subjective and/or exclusively holistic assessment at the workplace and to clarify and specify the key competences. Nevertheless, we realise that in the next step the compromise between a practical and feasible approach and the real clinical/medical context needs to be dealt with. In a follow up study, we will try to aggregate items in order to cluster competences that are clustered in the real clinical context as well. Next, the value of the inventory will be further investigated by validating the content of portfolios in different settings: by working together with several medical schools from abroad, the generalisability of our inventory will be tested.

The limitations of this study need to be acknowledged. Notably the fact that this Delphi study was started up with the Flemish version of the CanMEDS roles [22]. As this is not an exact translation of the original CanMEDS – presumably the difference in cultural identity makes this impossible – it could be argued that generalisation and usability in an international context is limited. Accordingly, we consider the development of the inventory at a single medical school as a limitation of this study. Besides, the experts might have kept in mind one type of portfolio in a specific clinical setting while participating in the Delphi process. Finally, the fact that the faculty members approved to introduce the CanMEDS framework when reforming the curriculum, could be seen as a bias.

Nevertheless, this study did not intend to support neither to criticise or disconfirm the CanMEDS. Rather we encouraged the experts to have a closer look at the CanMEDS roles and key competences with respect to workplace based assessment at an undergraduate level. We also selected experts with international expertise and all the experts were informed about the purpose of the study and were encouraged to have an open view. Additionally, the fact that experts scored the relevance differently in the second Delphi round as compared to the first, supports the efficacy of a Delphi procedure. In other words, respondents effectively took into account opinions of their colleagues, and if felt necessary, modified their own first opinion in a safe and anonymous environment without peer pressure. In this respect, our results and the developed inventory may be relevant to other institution who are working or plan to work with the CanMEDS Roles Framework.

## Conclusion

A competence inventory, starting from the CanMEDS competence framework, was developed using a Delphi procedure specifically focusing on assessment by a portfolio in clinical settings. This study has reinforced the importance and relevance of the CanMEDS roles but has also demonstrated the necessity of adapting such inventories in measurable and concrete items. If not, the applicability of the CanMEDS roles and key competences with regard to assessment at the workplace seems rather limited.

## Competing interests

The authors declare that they have no competing interests.

## Authors’ contributions

NM was the main investigator. She contributed on the study design, the data collection, the data analysis, and was the main manuscript writer. JD, ED, LVG, LB, and BDW contributed on the study design and manuscript corrections. BDW contributed on the data analysis. All the authors approved the final version of the manuscript.

## Pre-publication history

The pre-publication history for this paper can be accessed here:

http://www.biomedcentral.com/1472-6920/12/86/prepub

## Supplementary Material

Additional file 1: Table S1 The starting document for the 1^st^ Delphi round, based on the Flemish translation of the CanMEDS roles [23]. Click here for file
