# Supplementary material for: A Delphi study to construct a CanMEDS competence based inventory applicable for workplace assessment
Source: BMC Med Educ. 2012 Sep 14;12:86. doi: 10.1186/1472-6920-12-86 (PMC3599737; doi:10.1186/1472-6920-12-86)
Supplement: Additional file 1: Table S1 — The starting document for the 1st Delphi round, based on the Flemish translation of the CanMEDS roles [23]. [file 1472-6920-12-86-S1.docx]

**Additional file 1:** **The starting document for the 1^st^** **Delphi round, based on the Flemish translation of the CanMEDS roles** [23]

| **CanMEDS role** | **key competence** | **relevance** | | | **number of remarks** |
| --- | --- | --- | --- | --- | --- |
|  |  | **median** | **25-75 percentiles** | **% non-relevant scores** |  |
| **Medical Expert**  The student: | 1. possesses the required knowledge, insight/understanding and skills to develop a plan of care. | 5 | 4-6 | 12 | 11 |
|  | 2. can structure/perform a medical consultation (performs a relevant and accurate anamnesis, an appropriate physical examination; efficiently gathers, analyses, and interprets data of patients; generates diagnoses, and considers possible management plans). | 6 | 5-6 | 4 | 6 |
|  | 3. takes into account the prevention and follow up care of the patient. | 5 | 5-6 | 12 | 5 |
|  | 4. recognises specific illnesses. | 5 | 4.5-6 | 12 | 8 |
|  | 5. gathers the required information for composing patient’s medical records (addresses relevant questions, systematically searches in literature, critically evaluates literature to optimise decision making). | 6 | 5-6 | 8.6 | 8 |
|  | *General remark on the role of medical expert.* |  |  |  | 23 |
| **Communicator**  The student: | 6. performs an appropriate patient intake and anamnesis. | 6 | 5-6 | 4 | 6 |
|  | 7. can write down the patient’s medical record including adequate conclusions and/or questions about/on diagnosis, treatment, revalidation and palliation. | 5 | 5-6 | 8 | 3 |
|  | 8. can communicate his or her scientific research. | 5 | 4-5 | 4 | 9 |
|  | 9. can report verbally the patient’s medical record including appropriate conclusions and/or questions about diagnosis, treatment, revalidation and palliation. | 5 | 4-5 | 20 | 6 |
|  | *General remark on the role of communicator.* |  |  |  | 24 |
| **Collaborator**  The student: | 10. discusses with other health professionals or care providers; knows their profile and competences and recognises the opportunities of the different medical disciplines. | 5 | 3.5-6 | 24 | 7 |
|  | 11. is open-minded for additional information of the patient and his family. | 5 | 4-6 | 16 | 5 |
|  | *General remark on the role of collaborator.* |  |  |  | 15 |
| **Manager**  The student: | 12. organises his work in balance with his personal development. | 5 | 3.25-5.75 | 25.1 | 9 |
|  | 13. has insights/understanding in administrative tasks (in general practice and hospitals). | 4 | 3-5 | 32 | 4 |
|  | 14. can rank/classify information in order of importance and can prioritise responsibly. | 5 | 4-6 | 12 | 7 |
|  | 15. has a good notion of the health care system in Belgium and the financial-economical implications of health care;  learns how to deal efficiently with investments/fundings. | 4 | 3-5.25 | 28 | 6 |
|  | 16. uses information technology and gains insights in databases, both medical and patient related. | 5 | 4-5.75 | 12.5 | 9 |
|  | *General remark on the role of manager.* |  |  |  | 12 |
| **Health advocate**  The student: | 17. recognises psycho-sociological, economical, and biological aspects influencing patients’ health. | 5 | 4-6 | 16.7 | 5 |
|  | 18. is willing to sensitise patients’ awareness regarding health aspects. | 5 | 4-6 | 9 | 7 |
|  | 19. addresses the accessibility of health care, especially for vulnerable patients groups. | 5 | 4-6 | 21.7 | 5 |
|  | 20. knows juridical implications of health care (patient rights, professional secrecy or professional confidentiality, DNR-codes, end-of-life coaching ,…). | 5 | 4-6 | 12.5 | 5 |
|  | 21. learns to deal with incidents in practice and learns how to prevent them. | 5 | 4-6 | 13.6 | 8 |
|  | *General remark on the role of health advocate.* |  |  |  | 11 |
| **Scholar**  The student: | 22. poses relevant research questions and efficiently performs searches. | 5 | 3.5-6 | 25 | 7 |
|  | 23. questions the quality of medical scientific information sources and critically evaluates them. | 5 | 4-5 | 8.4 | 5 |
|  | 24. analyses his personal learning plan, has an adequate learning style, and self-evaluates or evaluates with peers his learning results. | 5 | 4-6 | 12 | 6 |
|  | 25. promotes the transfer of patient information between health care providers. | 4 | 2.75-5 | 36.3 | 11 |
|  | *General remark on the role of scholar.* |  |  |  | 9 |
| **Professional**  The student: | 26. cares for his patient in an integral and ethically correct way. | 6 | 5.5-6 | 4 | 2 |
|  | 27. knows professional, ethical and legal codes (and practices these). | 5 | 4.25-6 | 16.7 | 4 |
|  | 28. can cope with a multicultural society and context. | 5 | 4.5-6 | 4 | 3 |
|  | 29. can handle societal evolutions in health care. | 5 | 4-6 | 16.7 | 3 |
|  | 30. knows the limits of his own competences and acts accordingly. | 6 | 5-6 | 8.3 | 8 |
|  | 31. recognises ethical dilemmas. | 5 | 4.25-6 | 4.2 | 4 |
|  | 32. recognises unprofessional behaviour of himself and others. | 5 | 4.25-6 | 8.3 | 4 |
|  | *General remark on the role of professional.* |  |  |  | 10 |
| **general list** |  |  |  |  | 17 |

The first 2 columns represent the CanMEDS roles and key competences. The third column shows the median of the relevance and its 25–75 percentiles scored by the experts in the 1^st^ Delphi round and the percentage of scores ≤ 3 on relevance (% non-relevant scores). The last column displays the number of remarks made by the experts

highest median score (6)

lowest median score (4) + highest percentages of non-relevant scores
